# Protoplast transformation as a potential platform for exploring gene function in *Verticillium dahliae*

**DOI:** 10.1186/s12896-016-0287-4

**Published:** 2016-07-26

**Authors:** Latifur Rehman, Xiaofeng Su, Huiming Guo, Xiliang Qi, Hongmei Cheng

**Affiliations:** Biotechnology Research Institute, Chinese Academy of Agricultural Sciences, Beijing, 100081 China

**Keywords:** *Verticillium dahliae*, Driselase, Transformation, siRNAs

## Abstract

**Background:**

Large efforts have focused on screening for genes involved in the virulence and pathogenicity of *Verticillium dahliae*, a destructive fungal pathogen of numerous plant species that is difficult to control once the plant is infected. Although *Agrobacterium tumefaciens*-mediated transformation (ATMT) has been widely used for gene screening, a quick and easy method has been needed to facilitate transformation.

**Results:**

High-quality protoplasts, with excellent regeneration efficiency (65 %) in TB3 broth (yeast extract 30 g, casamino acids 30 g and 200g sucrose in 1L H_2_0), were generated using driselase (Sigma D-9515) and transformed with the GFP plasmid or linear GFP cassette using PEG or electroporation. PEG-mediated transformation yielded 600 transformants per microgram DNA for the linear GFP cassette and 250 for the GFP plasmid; electroporation resulted in 29 transformants per microgram DNA for the linear GFP cassette and 24 for the GFP plasmid. To determine whether short interfering RNAs (siRNAs) can be delivered to the protoplasts and used for silencing genes, we targeted the *GFP* gene of Vd-GFP (*V. dahliae* GFP strain obtained in this study) by delivering one of four different siRNAs—19-nt duplex with 2-nt 3′ overhangs (siRNA-gfp1, siRNA-gfp2, siRNA-gfp3 and siRNA-gfp4)—into the Vd-GFP protoplasts using PEG-mediated transformation. Up to 100 % silencing of *GFP* was obtained with siRNA-gfp4; the other siRNAs were less effective (up to 10 % silencing). *Verticillium* transcription activator of adhesion (*Vta2*) gene of *V. dahliae* was also silenced with four siRNAs (siRNA-vta1, siRNA-vta2, siRNA-vta3 and siRNA-vta4) independently and together using the same approach; siRNA-vta1 had the highest silencing efficiency as assessed by colony diameter and quantitative real time PCR (qRT-PCR) analysis.

**Conclusion:**

Our quick, easy transformation method can be used to investigate the function of genes involved in growth, virulence and pathogenicity of *V. dahliae*.

**Electronic supplementary material:**

The online version of this article (doi:10.1186/s12896-016-0287-4) contains supplementary material, which is available to authorized users.

## Background

*V. dahliae*, the causal agent of *Verticillium* wilt, is one of the most destructive plant pathogens, affecting over 400 plant species, including important ornamental, horticultural, agronomical and woody plants [1, 2]. Its control is difficult because it produces microsclerotia, which can survive in soil for several years [3]. Moreover, no effective fungicides or other chemicals are available to overcome this pathogen once the plant is infected. Despite a great deal of research, the molecular mechanisms behind the pathogenicity of this fungus have remained unclear [1].

An efficient transformation system is necessary for genetic manipulation and functional genomics studies of fungi [4]. A number of methods, *Agrobacterium tumefaciens*-mediated transformation (ATMT), PEG-mediated transformation, electroporation, and particle bombardment, have been used to transform different fungal species, including *V. dahliae*, with variable efficiencies [5–7]. ATMT is widely used for transforming various materials such as protoplasts, spores or hyphae of several fungal species [8, 9]. In *V. dahliae*, ATMT has been used for targeted gene disruption [7, 10–13] or deletion [14–16], but it is laborious and time-consuming. PEG-mediated transformation of *V. dahliae* was first reported in 1995 and has yielded a high efficiency of transformation [17–19]; however, obtaining the large amounts of high-quality protoplasts crucial to the success of the method is difficult for many fungal species including *V. dahliae*. Moreover PEG-mediated transformation results in a high percentage of transient transformation. Yet for filamentous fungi, this method has been ideal because it is simple and fast [20].

RNA interference (RNAi) is an effective tool to investigate gene function in various organisms [21–24]. In filamentous fungi, plasmid constructs expressing dsRNA have been applied for this purpose with a silencing efficiency between 70–90 % [25–28]. Although using this procedure for silencing gene is efficient, designing an RNAi plasmid is laborious. Synthetic siRNA can be used for the same purpose, e.g., incubation of synthetic siRNA with germinating spores of *A. nidulans* successfully silenced the target gene, leading to reduced mycelial growth [29]. In another study, synthetic dsRNA had in vitro antifungal activity against *adenylate cyclase*, *DNA polymerase alpha subunit* and *DNA polymerase delta subunit* in *F. oxysporum* and hindered spore germination [30]. However, transforming spores of certain fungal species like *V. dahliae* with siRNA is difficult as we have tried different ways to transform but obtained no satisfactory results (unpublished data). Thus, methods to directly transform protoplasts with synthetic siRNA to elucidate gene function have been sought. As reported for *Fusarium* sp. HKF15, siRNAs designed against hydroxymethyl glutaryl coenzyme A reductase (*hmgR*) and farnesyl pyrophosphate synthase (*fpps*) were used to transform protoplasts and knockdown these genes, however the silencing was effective for only 48 h [31].

Our main objective was to develop an easy and quick method to transform *V. dahliae* and facilitate screening of essential genes. First, we developed a protocol using one enzyme to obtain high quality protoplasts from *V. dahliae* with excellent regeneration efficiency in TB3 broth. We then compared variations in PEG-mediated transformation and electroporation methods to develop the most efficient protocol to transform the protoplasts with the GFP plasmid (circular and linear). We also used synthetic siRNAs (19-nt duplex with 2-nt 3′ overhangs) targeting the *GFP* gene in the GFP-transformed strain (Vd-GFP) and the *Vta2* gene, a regulatory gene that is essential for growth and conidiation of *V. dahliae* [16], in the wild-type strain (Vd-wt) using PEG-mediated transformation to test whether the siRNAs can enter the protoplasts and inhibit the expression of these genes. Our results indicated that PEG-mediated transformation is more effective than electroporation. Moreover the transformation efficiency for siRNAs and the linear GFP cassette was significantly higher than with the circular GFP plasmid.

Our method of protoplast isolation, regeneration and transformation has advantages over other available methods in its rapidity and ease for generating protoplasts using a single enzyme and transforming the protoplasts with high efficiency. These techniques are conducive for the study of gene function using siRNA silencing or gene deletion in a short period of time.

## Methods

### Fungal growth and spore harvesting

Strain V991 of *V. dahliae*, a highly toxic and defoliating wild-type pathogenic strain, provided by Prof. Guiliang Jian of the Institute of Plant Protection, Chinese Academy of Agricultural Sciences (CAAS), was cultured on PDA plates at 25 °C for 7-10 days. Sterile distilled water was added to the plates to harvest spores by gently scraping the agar with a sterile loop. The resulting suspension was filtered through a sterile 40 μm nylon filter (Falcon, REF352340) and centrifuged at 4000 rpm for 5 min. The final spore concentration was adjusted to 1.5 × 10^7^/mL.

### Protoplast isolation

Driselase (Sigma D-9515), selected after comparing with a variety of enzymes (cellulase: Sigma C1184; snailase: BBI SB0870; lysozyme: Sigma 62970), was prepared by dissolving 500 mg *driselase* in 25 ml NaCl (0.7 M) and centrifuged at 12,000 rpm for 10 min. The supernatant was taken and purified using 0.22 μm filters (MILLEX®GP). Two milliliters of *V. dahliae* spores (1.5 × 10^7^/ml) were cultured in 100 mL Complete Medium (CM: yeast extract 6 g, casein acid hydrolysate 6 g and 10 g sucrose in 1L H_2_0) for 16–24 h at 28 °C and 150 rpm. Mycelia were then separated from the culture and medium using a sterile 40 μm nylon filter, then washed 2–3 times with 0.7 M NaCl. The harvested mycelia were aseptically transferred to 10 ml of the driselase solution and incubated at 33 °C for 0.5–3.5 h at 60 rpm. The preparation was then checked every 30 min for protoplast release. After the incubation time, the mixture was then filtered using a sterile 40 μm nylon filter to remove any hyphal fragments, and the protoplasts were centrifuged at 2800 rpm for 5 min. The supernatant was discarded, and the pellet was washed 2–3 times either with 1 M sorbitol, in case the protoplast has to be used for electroporation, or with STC buffer (20 % sucrose, 10 mM Tris-HCl pH 8.0, and 50 mM CaCl_2_), if used for PEG-mediated transformation. The concentration of protoplasts was adjusted with either 1 M sorbitol or STC to 10^6^/ml.

### Regeneration of protoplasts

The ability of the protoplasts to regenerate was examined in CM, TB3 and Czapek-Dox broths. Briefly, 200 μl protoplasts (10^6^/ml) were cultured in 5 ml broth and incubated at 25 °C for 18 h. Protoplasts were observed for regeneration with a light microscope at 20× magnification, and the percentage of regeneration was calculated by counting the number of regenerated protoplasts out of total protoplasts cultured. In order to isolate a single colony, the regenerated protoplast suspension was centrifuged at 2800 rpm, the supernatant discarded and pellet was resuspended in 200 μl CM broth, serially diluted and cultured on PDA for 5–7 days until the colonies appeared.

### GFP plasmid and siRNAs

The GFP plasmid (pCH-sGFP, Additional file 1) was kindly provided by Professor Xie Bingyan of the Institute of Vegetables and Flowers, CAAS. Primers GFP-1 5′ CTTTCGACACTGAAATACGTCG3′ and GFP-2 5′ GCATCAGAGCAGATTGTACTGAGAG3′ were used to amplify the GFP cassette from the GFP plasmid.

The siRNAs targeting different regions of the *GFP* gene (siRNA-gfp1, siRNA-gfp2, siRNA-gfp3 and siRNA-gfp4) and the *Vta2* gene (siRNA-vtaNC, siRNA-vta1, siRNA-vta2, siRNA-vta3 and siRNA-vta4) were designed and synthesized by Oligobio, Beijing, China. The sequences of siRNAs are given in Table 1 and the positions of these siRNAs along the genes are shown in Additional file 1 and Additional file 2.

**Table 1:** siRNA sequences developed against *GFP* and *Vta2*

Name	Sense sequence	Antisense sequence
siRNA-gfp1	UCUUCAAGGACGACGGCAATT	UUGCCGUCGUCCUUGAAGATT
siRNA-gfp2	GCCACAACGUCUAUAUCAUTT	AUGAUAUAGACGUUGUGGCTT
siRNA-gfp3	GCAUGGACGAGCUGUACAATT	UUGUACAGCUCGUCCAUGCTT
siRNA-gfp4	UCAAGGAGGACGGCAACAUTT	AUGUUGCCGUCCUCCUUGATT
siRNA-vta1	CCAGGGCAUGUACUCUCAATT	UUGAGAGUACAUGCCCUGGTT
siRNA-vta2	GCAUGUACUCUCAACACAATT	UUGUGUUGAGAGUACAUGCTT
siRNA-vta3	CCACGCUCAACACCUCUAUTT	AUAGAGGUGUUGAGCGUGGTT
siRNA-vta4	GGCGCAACAAGCAAGCAAUTT	AUUGCUUGCUUGUUGCGCCTT
siRNA-vtaNC	UUCUCCGAACGUGUCACGUTT	ACGUGACACGUUCGGAGAATT

### PEG-mediated transformation

For PEG-mediated transformation, an established protocol was followed with some modifications [32]. Briefly, 200 μl protoplasts (10^6^/ml) was mixed with 12 μg GFP plasmid (12.2 kb, bearing the hygromycin resistance cassette (hph) as a selection marker) or linear GFP cassette (3.3 kb) in a 50 ml Falcon tube and incubated on ice for 30 min; 1.5 ml 60 % PEG solution in STC buffer was added to the tube dropwise, gently swirled and left at room temperature for 15 min followed by the addition of 5 ml TB3 broth. The tubes were incubated at 25 °C for 18 h, and *GFP* expression was checked with a fluorescence microscope (Zeiss Axio Imager M1, Jena, Germany). Transformants, transformed with GFP plasmid, were selected on PDA media supplemented with hygromycin B (50mg/mL final concentration) after regeneration in TB3 broth. Further confirmation of the positive transformants was made by PCR using GFP-CF 5′AGCTGGACGGCGACGTAAAC3′ and GFP-CR 5′GATGGGGGTGTTCTGCTGGT3′ primers.

### Electroporation

Before using electroporation for transformation, protoplasts were shocked at different field strengths from 100-1000 V/cm to ensure that electroporation had no or a very low lethal effect on the regeneration of protoplasts.

The protoplasts were mixed with 12 μg of the GFP plasmid or linear GFP cassette as described above, using 1 M sorbitol as the buffer, and kept on ice for 10 min. The mixtures were transferred to a 0.2-cm gap cuvette (BioRad, Hercules, CA), and different voltages (300, 400 and 500 V) were applied for 5 ms using a GenPulser Xcell electroporation system (BioRad). Immediately after electroporation, the protoplasts were transferred to 5 ml TB3 broth and incubated at 25 °C for 18 h. After regeneration of protoplasts, the culture was centrifuged at 2800 rpm for 5 min. The supernatant was discarded, and the pellet was resuspended in 200 μl TB3 and observed with a fluorescence microscope.

### siRNA inhibition assay for *GFP* and *Vta2* genes

For targeting the *GFP* gene, 200 μl Vd-GFP protoplasts (10^6^/ml) were transformed with 10 μM siRNA-gfp1, siRNA-gfp2, siRNA-gfp3 or siRNA-gfp4 by PEG-mediated transformation and regenerated for 18 h as described. Inhibition of *GFP* expression was checked using fluorescence microscopy by counting the number of hyphae with fluorescence. The percentage of *GFP* inhibition was determined by dividing the number of hyphae with no fluorescence on the number of hyphae with fluorescence multiplied by 100.

Similarly, for silencing *Vta2* gene, protoplasts obtained from Vd-wt were treated with siRNA-vta1, siRNA-vta2, siRNA-vta3 and siRNA-vta4 independently in a final concentration of 10 μM or with 2.5 μM siRNAs mix using PEG-mediated transformation in RNase free environment. siRNA-vtaNC was used as a negative control. Briefly, protoplasts obtained from vd-wt (10^6^/ml) were mixed with either of the siRNAs or with mixed siRNAs in 50 mL falcon tube and incubated on ice for 30 min; 1.5 ml 60 % PEG solution in STC buffer was added to the tube dropwise, gently swirled and left at room temperature for 15 min followed by the addition of 5 ml TB3 broth. After 18 h of incubation in TB3 broth, the cultures were centrifuged at 2500 rpm for 5 min, the supernatant was discarded, and the pellet was resuspended in 200 μl TB3 broth and pipeted onto the center of CM agar plates. The colony diameter was measured after 10 days at 25 °C.

### qRT-PCR analysis of *Vta2* expression level

In order to further confirm that the reduction in growth of *V. dahliae* was due to silencing of *Vta2* gene, we conducted qRT-PCR. After treating the protoplasts with siRNAs (siRNA-vtaNC, siRNA-vta1, siRNA-vta2, siRNA-vta3, siRNA-vta4 and siRNA-vtamix respectively), they were cultured in TB3 broth for 72 h and the mycelia were harvested for RNA extraction by RNA Extraction Kit (YPHBio, Tianjin, China). qRT-PCR was carried out in 7500 Real Time PCR System (ABI, Massachusetts, USA). Gene specific primers were used: vta2-F 5′GGCTTCCTCAAGGTCGGCTATG3′, vta2-R 5′GCTGCATGTCATCCCACTTCTTC3′, Vdactin-F 5′GGCTTCCTCAAGGTCGGCTATG3′ and Vdactin-R 5′GCTGCATGTCATCCCACTTCTTC3′ *Vdactin* was used as a housekeeping gene [33]. The relative expression of the targeted gene was analyzed using the 2^-∆∆Ct^ method. The standard curve met experimental requirements (*R*^2^ > 0.99, E > 95 %) [34].

### Statistical analysis

All experiments were done in three independent replicates. Means ± standard deviation and significant differences were determined using Duncan’s multiple range test and *t*-test with *p*-values < 0.05 in SPSS 17.0 software (SPSS Inc., Chicago, IL, USA).

## Results

### Isolation and regeneration efficiency of protoplasts

In order to select an efficient enzyme for protoplasts isolation from *V. dahliae*, we treated the mycelia with different enzymes (driselase, cellulase, snailase and lysozyme) and found that driselase resulted in maximum protoplasts yield (Additional file 3). While investigating the effect of driselase concentration on the yield of protoplast, 20 mg/mL was found the best (Additional file 3).

**Fig. 1:**
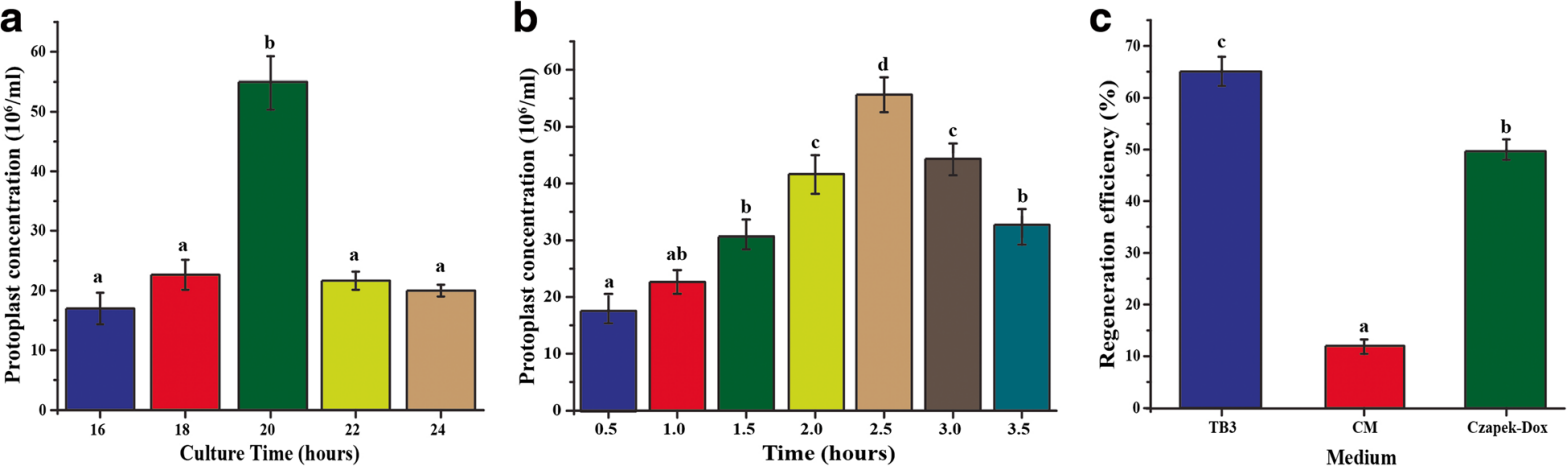
Optimization of protoplast isolation and regeneration. **a** Protoplast isolation efficiency from mycelia cultured for 16 to 24 h and then treated for 2.5 h in 10 ml driselase mixture. **b** Protoplast isolation efficiency after various digestion times with driselase. Mycelia were harvested at 20 h post inoculation. **c** Regeneration efficiency in different media. Protoplasts (200 μl of 10^6^/ml) were cultured in 5 ml TB3, CM or Czapek-Dox broth. After 18 h, the regeneration efficiency was measured as the number of protoplast regenerated out of total number of protoplast cultured multiplied by 100

For selecting the optimal mycelial age and enzymolytic time to isolate protoplasts, spores were cultured for different times (16, 18, 20, 22 and 24 h) and then treated with driselase (0.5–3.5 h). The optimal mycelial age was found to be 20 h which yielded 5.5 × 10^7^/ml ± 0.275 protoplasts when treated with driselase (Fig. 1a), while 2.5 h enzymolysis time was observed to produce the maximum number of protoplasts for all culture ages (Fig. 1b). When the three media were tested for regeneration efficiency, the efficiency was highest in TB3 (65 ± 3 %) (Fig. 1c).

### Observation of fluorescence from *GFP* expression

**Fig. 2:**
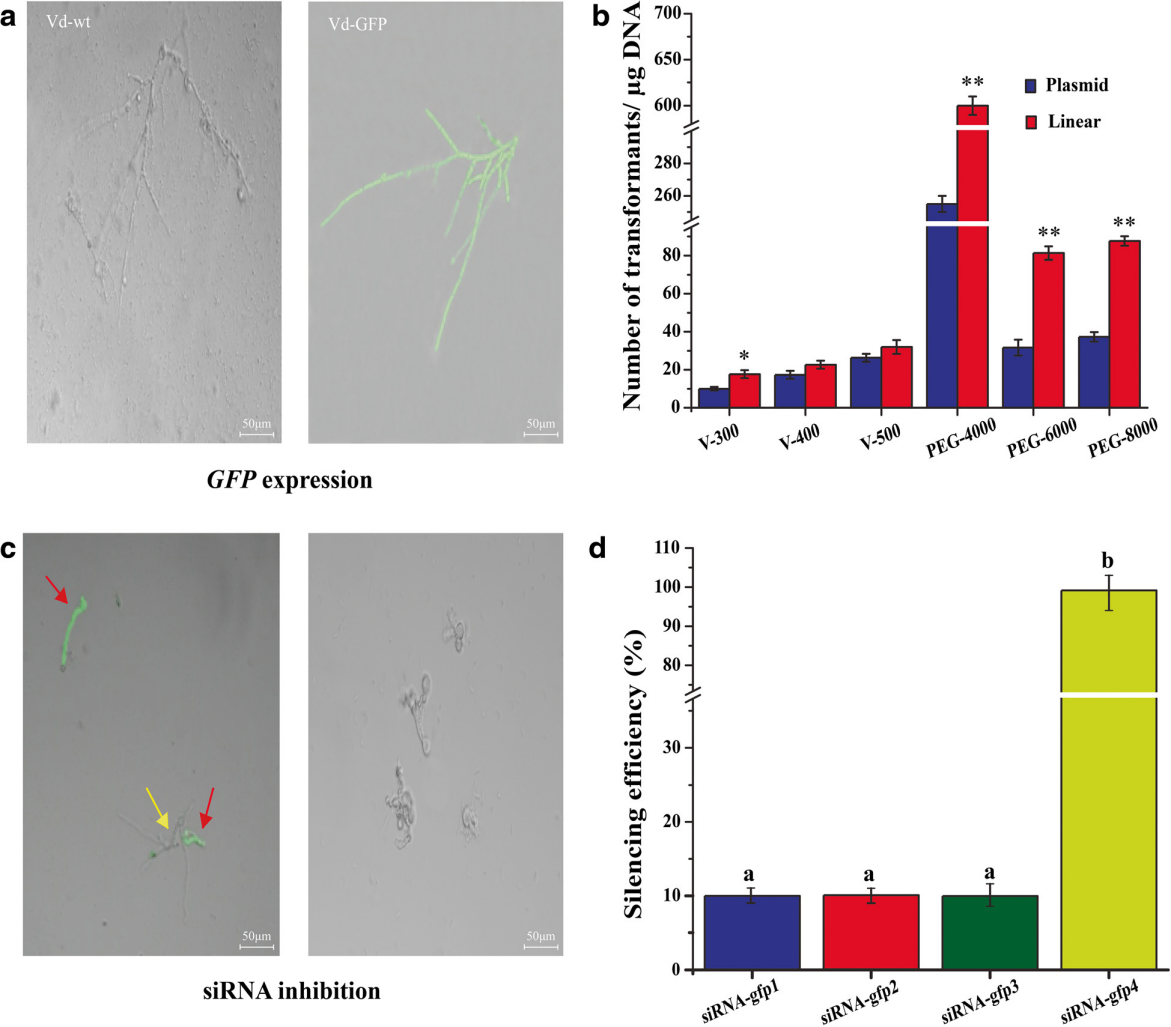
Fluorescence detection of GFP expression in hyphae regenerated from transformed protoplast of *V. dahliae*. **a** GFP expression after 18 h of incubation. Protoplasts (200 μl of 1 × 10^6^ /ml) were transformed with 12 μg of either GFP plasmid or linear GFP cassette and cultured. Fluorescence was observed in hyphae regenerated from transformed protoplast of V. dahliae after 18 h incubation in TB3. **b** Transformation efficiency of protoplasts using electroporation (300-500 V) or PEG-mediated transformation (PEG-4000, 6000 and 8000). After transformation, the protoplasts were cultured in TB3 broth for 18 h. Number of transformants was calculated per microgram DNA by counting the number of hyphae with GFP fluorescence. **c** Silencing of GFP expression with siRNA. Vd-GFP protoplasts were transformed with 10 μM of 4 different siRNAs (siRNA-gfp1, siRNA-gfp2, siRNA-gfp3 and siRNA-gfp4) separately by PEG-mediated transformation. The regenerated mycelia from the transformed protoplasts were observed for GFP fluorescence. **d** Assay for siRNA inhibition of GFP. Inhibition of GFP expression by siRNA-gfp1, siRNA-gfp2, siRNA-gfp3 and siRNA-gfp4 was compared after in the regenerated hyphae from the transformed protoplasts

Soon after PEG-mediated transformation and electroporation, the protoplasts were cultured for 18 h in TB3 broth to detect *GFP* expression as an indicator of transformation. Strong GFP fluorescence was observed in the transformed protoplasts, but none was seen in the Vd-wt (Fig. 2a). PEG-4000 gave the highest transformation of all the methods for both linear GFP (600 ± 20 transformants/μg DNA) and for GFP plasmid (250 ± 10 transformants/μg DNA) (Fig. 2b). Electroporation for both GFP plasmid (24 ± 1 transformants/μg DNA) and linear GFP cassette (29 ± 1 transformants/μg DNA) was significantly lower than the PEG-mediated (PEG-4000) transformation.

### GFP transformants selection and stability of the transgene

GFP transformants derived from GFP plasmid transformation were selected after 5–7 days of culturing the regenerated protoplasts on PDA plates supplemented with hygromycin B. The selected GFP transformants fluoresced strongly when viewed with fluorescence microscopy. Further confirmation of the transformants was made by PCR (Additional file 4). Expression of GFP was observed from three generations of transformants, indicating stable *GFP* expression throughout these three generations.

### Silencing of *GFP* gene in strain Vd-GFP with siRNAs

Protoplasts of strain Vd-GFP were treated with the different siRNAs targeting the *GFP* gene, and checked for inhibition of *GFP* expression after regeneration (Fig. 2c). We found out that siRNA-gfp4 gave the best silencing efficiency (up to 100 %) compared with 10 % or less with siRNA-gfp1, siRNA-gfp2 and siRNA-gfp3 (Fig. 2d). The silencing of the *GFP* gene lasted for at least 72 h.

### Silencing of *Vta2* gene

**Fig. 3:**
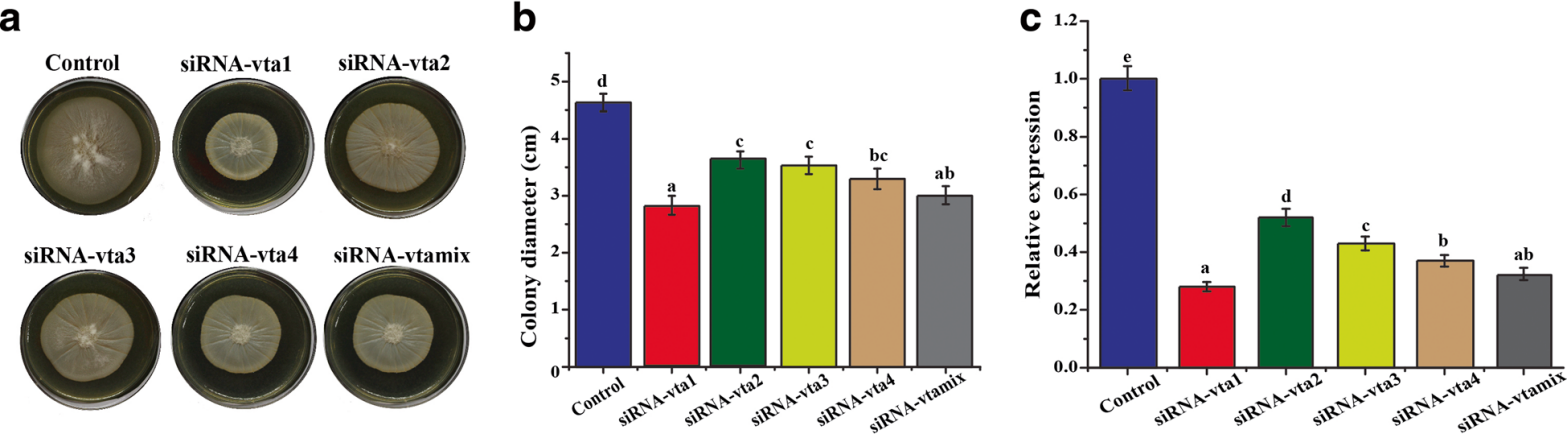
Assay for siRNA inhibition of *Vta2* gene. Protoplasts (200 μl of 10^6^/ml) isolated from Vd-wt were transformed with 10 μM siRNA-vta1, siRNA-vta2, siRNA-vta3 and siRNA-vta4 independently in separate tubes and also with 2.5 μM each of these siRNAs via PEG-mediated transformation. Protoplasts were regenerated for 18 h in TB3 broth at 25 °C, pelleted at 2500 rpm for 5 min, then resuspended in 200 μl TB3 broth and cultured in the center of CM agar plate. Colony diameter was measured for each group after 7 days at 25 °C (Control, siRNA-vta1, siRNA-vta2, siRNA-vta3, siRNA-vta4 and siRNA-vtamix). **a** Colony morphology of different groups on CM agar. **b** Colony diameters of control and siRNA groups. **c** Relative expression level of *Vta2* gene in different siRNA treated groups. RNA was isolated from mycelia harvested after 72 h, first strand cDNA was synthesized and qRT-PCR was conducted for different siRNA groups

To validate whether *V. dahliae* genes can be silenced by siRNA using *Vta2* as a reference gene, the gene was successfully silenced with siRNAs. On CM plates, the colony diameter of siRNA-vta1 group (2.8 cm) was significantly smaller than that of the siRNA-vtaNC group (4.6 cm) (Fig. 3a and b). The colony diameters of siRNA-vta2, siRNA-vta3, siRNA-vta4 and siRNA-vtamix groups were 3.6 cm, 3.5 cm, 3.2 cm and 3.0 cm, respectively. As determined by colony diameter, siRNA-vta1 had the best silencing efficiency. To further confirm whether the reduction in colony diameter was due to silencing of *Vta2* gene, qRT-PCR was conducted to determine the relative expression level of this gene in all the groups. The data was in accordance with that obtained from colony assessment. Expression level of *Vta2* in siRNA-vta1 group was significantly lower than the other groups (Fig. 3c).

## Discussion

In this study, we isolated and regenerated protoplasts from *V. dahliae*, then transformed the protoplasts with the GFP plasmid and linear GFP cassette using PEG-mediated transformation and electroporation, and silenced the *GFP* and *Vta2* genes using siRNAs.

Protoplasts have been isolated from many fungal species at various efficiencies depending on the species and conditions. Driselase has proved efficient for protoplast isolation from *Fusarium graminearum* (ca 10^9^ g^-1^ wet mass) and *Ascosphaera apis* (98.36 × 10^5^ mL^-1^ of protoplasts) [35, 36]. For *V. dahliae*, protoplasts were previously isolated using a combination of two enzymes [18, 19]. In our study, we isolated protoplasts from *V. dahliae* by using a single enzyme *driselase*, with up to 90 % efficiency. The main differences in our protocol and that developed in the previous study [19] are the number of spores they cultured for protoplasts isolation and the enzymolysis temperature. In our study 1.5 × 10^7^/ml spores were cultured for obtaining mycelia to be digested with the enzyme while they used 10^6^/ml. We used 33 °C as the optimum enzymolysis temperature and 20 h old culture the best for protoplast isolations as compared to their 30 °C and 24 h old culture. There is a difference in the media used for the growth of mycelia as well between the two protocols that can also have a significant effect on the protoplast production [36–39].

The efficiency of protoplast isolation, in addition to the digestion enzyme and other factors, also depends on the age of the mycelia. Young and exponentially growing mycelia are the best choices for protoplast isolation [36], but the optimal age of the mycelia varies for different species, e.g., 60 h for *Blakeslea trispora* and 2 days for *Pleurotus pulmonarius* and *Pleurotus florida* [40, 41]. For *V. dahliae*, previous studies have used 24-h-old mycelia for protoplast isolation [18, 19], but here we obtained more protoplasts from the 20-h culture than from the 24-h culture. The probable reason for this can be the sensitivity of the younger mycelia to the digesting enzymes, with the increase in growth the cell wall becomes thicker and the mycelia would be digested more difficultly leading to decreased protoplast yield [36, 38, 39]. In addition to the age of mycelia, the protoplast yield also depends on the duration of the enzyme digestion, which has ranged from 3 h to 16 h for maximum protoplast release in other fungal species [37, 40, 42]. The optimum time of enzymolysis in our study was 2.5 h. Less time is presumably not enough for all the mycelia to be digested by the enzyme, while prolonged enzymolyis can result in the breaking of protoplasts [37, 40–42].

Regeneration of protoplasts is a vital step and is the main limiting factor in a transformation experiment. Thus, a high frequency of regeneration is necessary for genetic manipulation of the particular fungus. Protoplasts from different fungi have been isolated with various regeneration frequencies, ranging from 3.3 to 77.5 % [37, 40–42], partly depending on the media used to culture the protoplast. For example, the protoplast regeneration efficiencies for *B. trispora* and *Nodulisporium sylviforme* were found to be 77.5 % and 72 % on PDA respectively while the regeneration frequencies decreased (for *B. trispora* 32.5 % on RM and for *N. sylviforme* 44 % on CM) with the use of other media [40, 42]. In our study, the frequency of regeneration was about 5-fold higher in TB3 broth than in CM broth.

To increase the transformation efficiency of *V. dahliae* protoplasts, we also tested a number of protocols to determine the best one. Transformation efficiency of various fungal species with PEG has been variable, e.g., 102 transformants/1 μg DNA for *V. fungicola*, 100-200 for the basidiomycete *Pleurotus ostreatus* [5, 43], and for *V. dahliae*, 10–50 transformants/1 μg DNA [18]. We obtained much higher yields in our experiments: 250 transformants/1 μg DNA using the GFP plasmid (12.2 kb) and 600 using the linear GFP cassette (3.3 kb) and PEG-4000. The higher frequency of transformation might be due to the high quality of protoplasts obtained. Plasmid size also plays a vital role in the transformation efficiency. Previous studies have indicated that increasing plasmid size results in decreased transformation efficiency [44, 45]. The fewer transformants obtained with the GFP plasmid in comparison to the linear GFP cassette is thus probably due to the large size of the plasmid. On the other hand, transforming *V. dahliae* protoplast using electroporation did not yield promising results for either of the GFP plasmids. The main hurdle in electroporation is the regeneration of the protoplasts. As the voltage increases, the regeneration capacity of the protoplast decreases. At low voltage (300–500 V), the transformation efficiency ranged from 10 to 29 transformants/1 μg DNA for GFP plasmid and linear GFP cassette, respectively, much lower than with PEG-4000.

RNAi is a powerful reverse genetics tool for deciphering gene function in various organisms including fungi [46]. Characterizing gene function using gene deletion, disruption or insertion is a time-consuming process. Downregulation of a gene using RNAi is an alternative method in functional genomics as it is a rapid process as compared to the deletion or disruption of a gene. Moreover this approach is particularly helpful in studying essential genes or genes present in multiple copies within the genome that could compensate for each other’s function. In fungi, synthetic siRNAs have been used to downregulate specific genes. For example, *hmgR* and *fpps* in *Fusarium* sp. HKF15 were silenced by delivering siRNAs designed against these genes into the protoplasts [31]. In another study, three siRNA sequences (Nor-Ia, Nor-Ib, Nor-Ic) targeting the mRNA sequence of the *aflD* gene were tested for controlling aflatoxin production in *Aspergillus flavus* and *Aspergillus parasiticus* [47]. Designing siRNAs that are more effective at downregulating is essential for gene silencing. Several siRNAs designed from different sites within the same gene can have striking differences in silencing efficiency [48, 49] as shown by the silencing of the *GFP* gene at various efficiencies using different siRNAs.

*Vta2* gene was used as a reference gene for siRNA inhibition assay because its inhibition can easily be assessed from the colony growth [16]. After treating protoplasts obtained from Vd-wt transformed with different siRNAs designed for this gene, we observed a significant decrease in the colony diameter of the siRNA-treated groups compared with the control group. Differences in colony diameter were also observed among the siRNA groups, indicating that siRNA designed from different locations within the gene can have strikingly different silencing effects. The difference in the colony diameter among the siRNA groups and the control groups lasted for about 10 days, sufficient time for characterizing a gene.

## Conclusion

Our improved method greatly increased the number of transformants per microgram of DNA over the others available. This method will be useful for elucidating gene functions by downregulating a particular gene of interest using siRNA and constructing gene deletion mutants of *V. dahliae* in a shorter time than required for ATMT.

## Abbreviations

ATMT, *Agrobacterium tumefaciens*-mediated transformation; CM, complete medium; PDA, potato dextrose agar; PEG, polyethylene glycol; siRNA, short interfering RNA; *Vta*, *Verticillium* transcription activator of adhesion

## Additional files

Additional file 1: Sketch of pCH-sGFP and position of siRNA along the *GFP* gene. (A) Diagram of GFP plasmid (pCH-sGFP). (B) Position of siRNAs along the *GFP* gene. siRNAs were designed and synthesized by Oligobio, Beijing, China. (JPG 9062 kb) Additional file 2: Position of siRNAs along the *Vta2* gene of *V. dahliae*. The position of different siRNAs designed to target this gene is shown in this figure. Sequence underlined with different colors shows different siRNAs. (JPG 7797 kb) Additional file 3: Selection of efficient enzyme and the effect of driselase concentration on the protoplasts isolation from *V. dahliae*. (A) Protoplasts isolation efficiency from the mycelia of Verticillium dahliae by treating with different enzymes, (B) The effect of driselase concentration on the release of protoplasts. (JPG 433 kb) Additional file 4: Confirmation of GFP transformants by PCR. Single colony was selected and cultured in CM for 5-7 days. Mycelia were harvested and genomic DNA was isolated. PCR was carried out with gene specific primers. (JPG 152 kb)
