# Supplementary figures and images for: Protoplast transformation as a potential platform for exploring gene function in Verticillium dahliae
Source: BMC Biotechnol. 2016 Jul 26;16:57. doi: 10.1186/s12896-016-0287-4 (PMC4960691; doi:10.1186/s12896-016-0287-4)

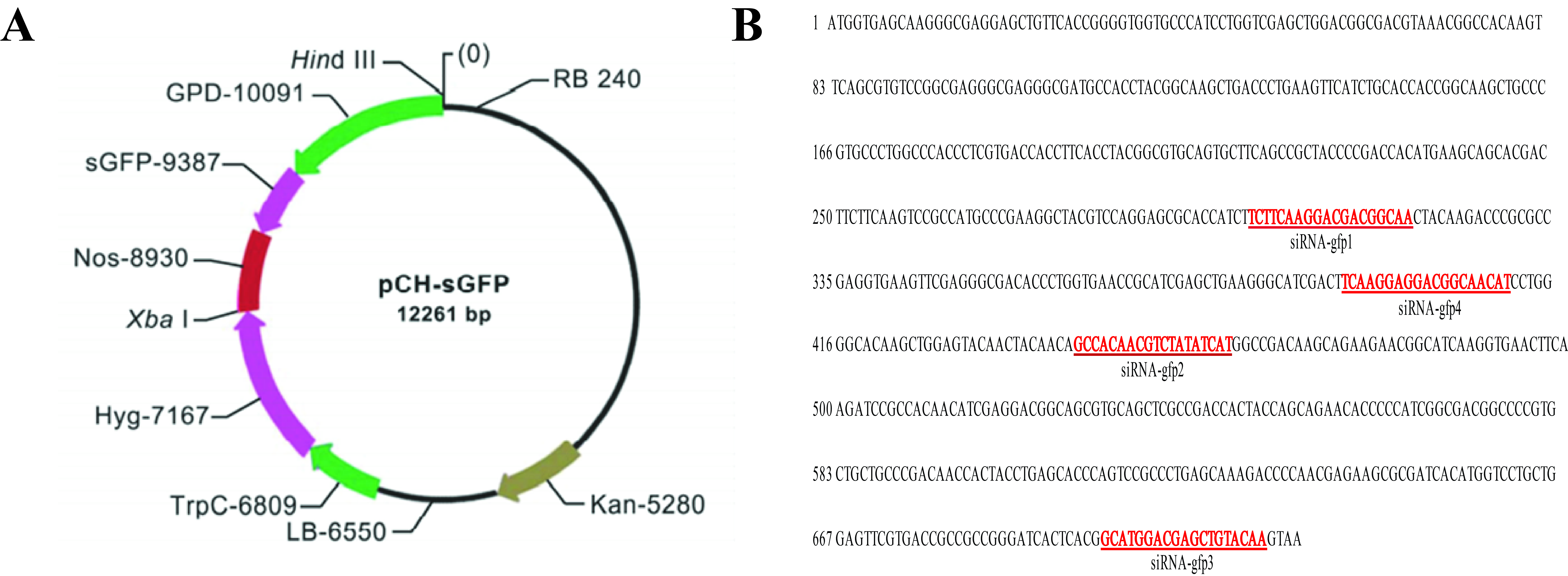

Supplement: Additional file 1: — Sketch of pCH-sGFP and position of siRNA along the GFP gene. (A) Diagram of GFP plasmid (pCH-sGFP). (B) Position of siRNAs along the GFP gene. siRNAs were designed and synthesized by Oligobio, Beijing, China. (JPG 9062 kb) [file 12896_2016_287_MOESM1_ESM.jpg]

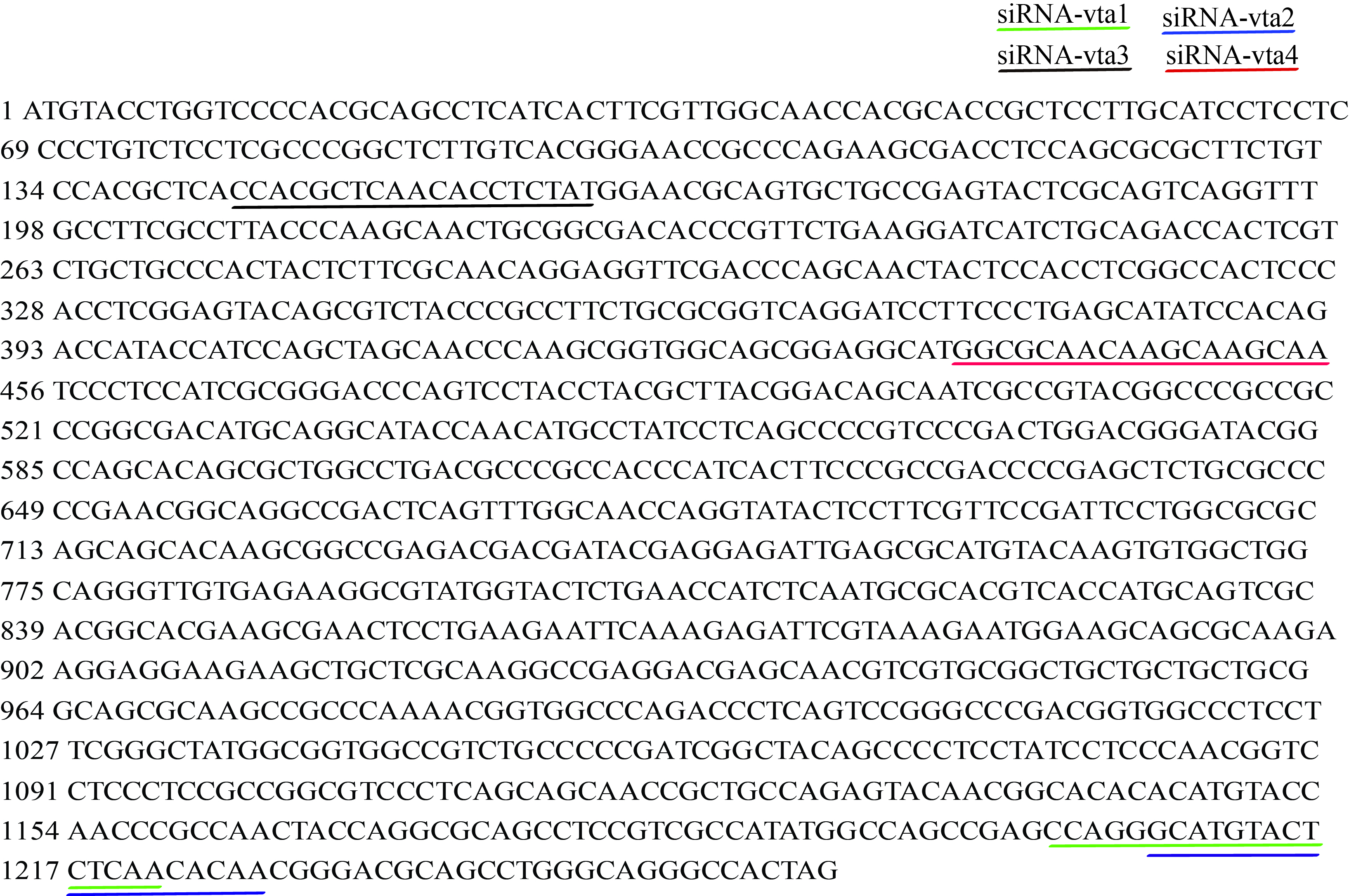

Supplement: Additional file 2: — Position of siRNAs along the Vta2 gene of V. dahliae. The position of different siRNAs designed to target this gene is shown in this figure. Sequence underlined with different colors shows different siRNAs. (JPG 7797 kb) [file 12896_2016_287_MOESM2_ESM.jpg]

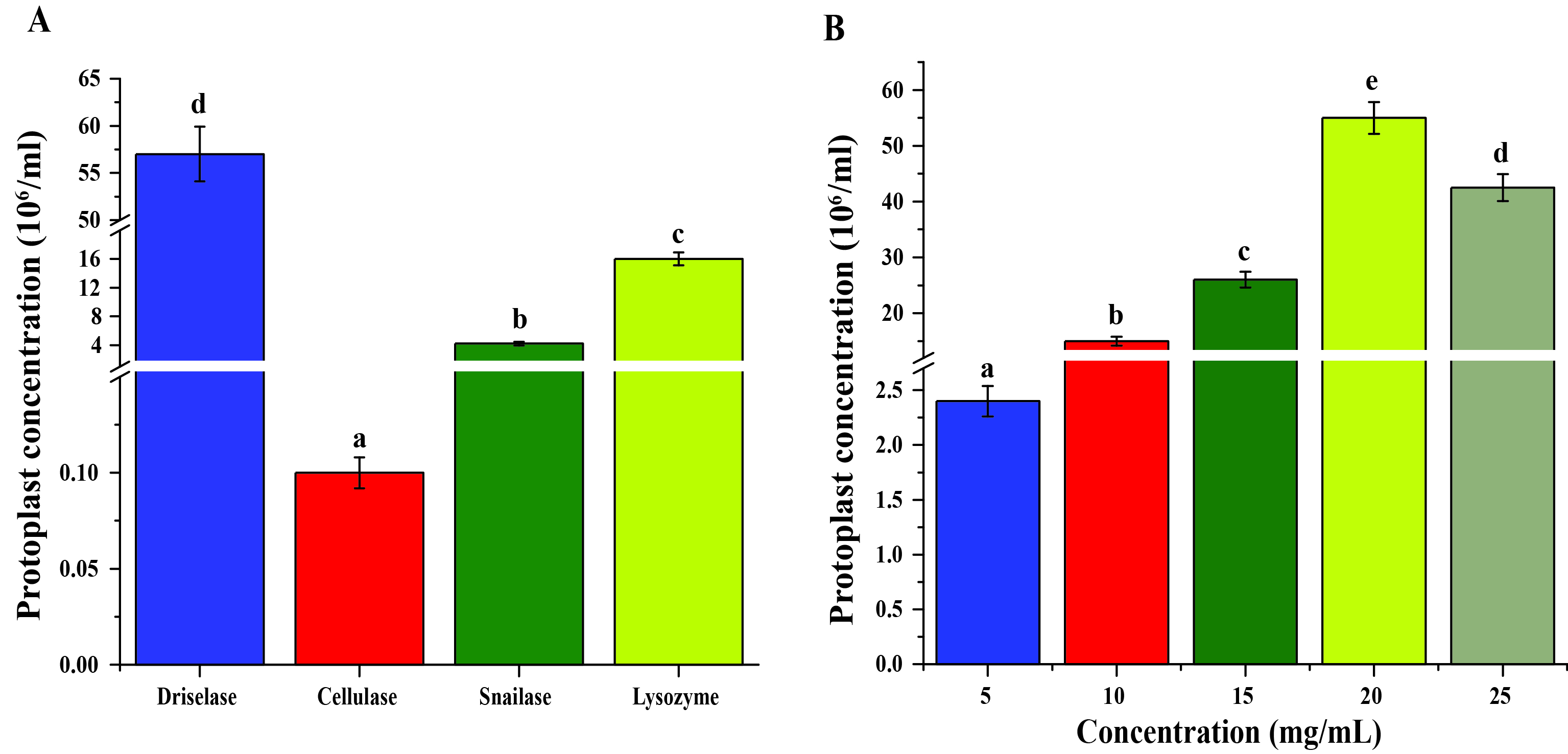

Supplement: Additional file 3: — Selection of efficient enzyme and the effect of driselase concentration on the protoplasts isolation from V. dahliae. (A) Protoplasts isolation efficiency from the mycelia of Verticillium dahliae by treating with different enzymes, (B) The effect of driselase concentration on the release of protoplasts. (JPG 433 kb) [file 12896_2016_287_MOESM3_ESM.jpg]

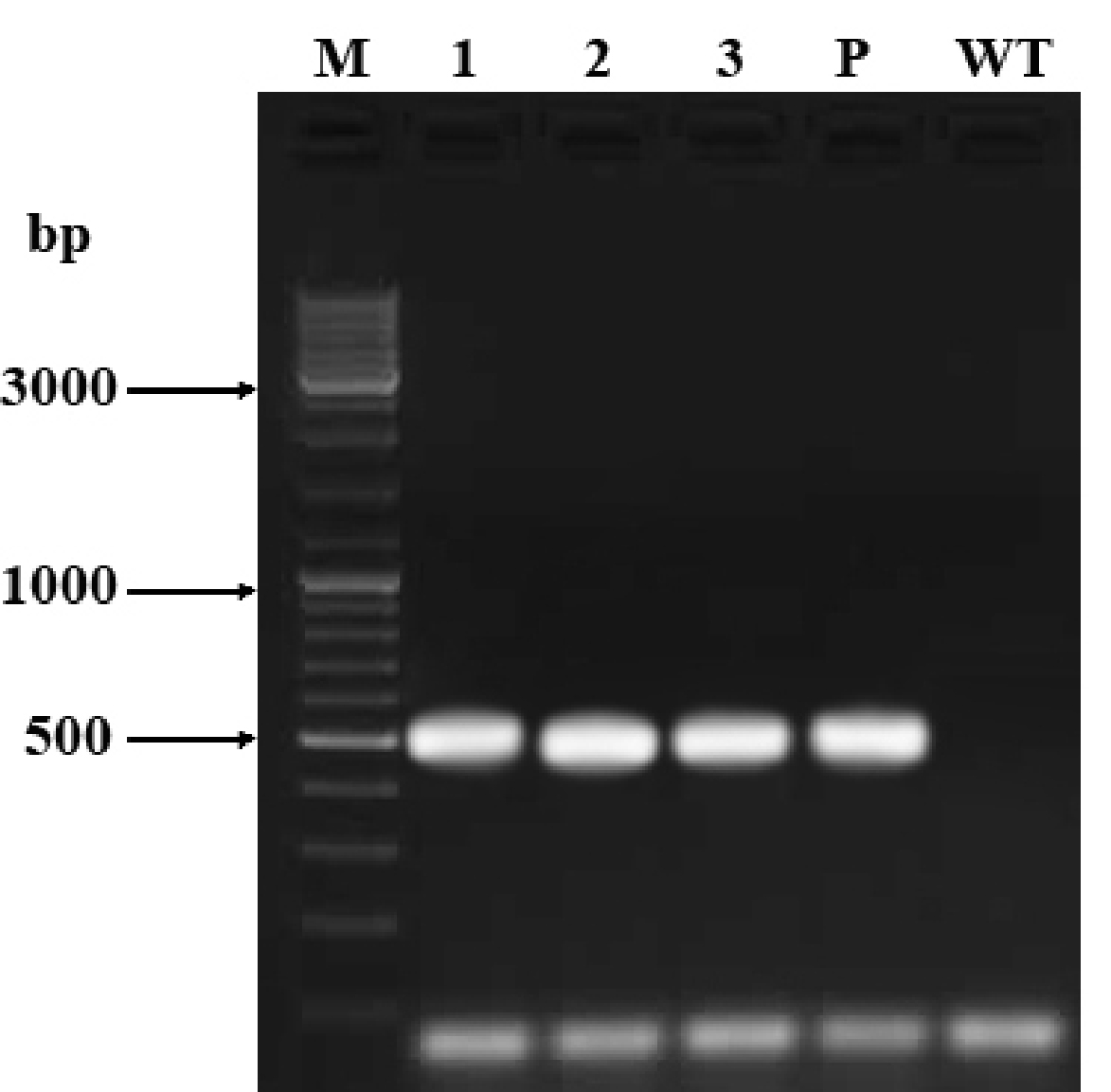

Supplement: Additional file 4: — Confirmation of GFP transformants by PCR. Single colony was selected and cultured in CM for 5-7 days. Mycelia were harvested and genomic DNA was isolated. PCR was carried out with gene specific primers. (JPG 152 kb) [file 12896_2016_287_MOESM4_ESM.jpg]
